# The mechanism regulating the cytotoxicity of γδ T cells activated by mycobacterium tuberculosis heat-resistant antigen based on RNA-seq analysis

**DOI:** 10.3389/fimmu.2026.1707304

**Published:** 2026-01-28

**Authors:** Yamin Song, Fangzheng Guo, Huiting Dai, Xiaoyu Zhou, Sihang Dong, Hui Zhang, Zhongqing Qian, Baiqing Li, Xiaojing Wang, Tao Xu, Hongtao Wang

**Affiliations:** 1 Anhui Province Key Laboratory of Immunology in Chronic Diseases, Laboratory Medicine Experimental Center, Laboratory Medicine College, Bengbu Medical University, Bengbu, China; 2 Department of Neurosurgery, The First Affiliated Hospital of Bengbu Medical University, Bengbu, China; 3 Anhui Province Key Laboratory of Respiratory Tumor and Infectious Disease, Molecular Diagnosis Center, The First Affiliated Hospital of Bengbu Medical University, Bengbu, China

**Keywords:** cytotoxicity, Mycobacterium tuberculosis heat-resistant antigen (Mtb-HAg), RNA sequencing, tuberculosis, γδ T cells

## Abstract

**Introduction:**

Tuberculosis (TB), caused by Mycobacterium tuberculosis (Mtb), remains a major global health threat. γδ T cells, critical innate immune responders, provide rapid anti-TB defenses and act as a bridge between innate and adaptive immunity. Studies have demonstrated that γδ T-cell activation by phosphoantigens is mediated by butyrophilin subfamily 3 member A1 (BTN3A1), leading to enhanced cytokine production and cytotoxicity. Mtb heat-resistant antigen (Mtb-HAg), extracted from Mtb H37Ra, specifically activates γδ T cells and induces cytokine secretion. However, the contribution of Mtb-HAg to γδ T cell-mediated cytotoxicity and its dependence on BTN3A1 remain unclear.

**Methods:**

This study explored the regulatory mechanism of Mtb-HAg on the cytotoxic function of γδ T cells through RNA-Seq analysis and functional validation methods. The RNA-Seq analysis to profile the transcriptome of Mtb-HAg-activated γδ T cells.The expression of key cytotoxic factors was analyzed using ELISA, and the capacity of activated γδ T cells to inhibit intracellular Mtb growth was assessed using a co-culture assay with Mtb-infected macrophages. The specific role of BTN3A1 was investigated using a blocking antibody to assess its impact on activation markers, cytotoxic factor secretion, and mycobacterial killing efficiency.

**Results:**

RNA-Seq analysis revealed that Mtb-HAg-activated γδ T cells are significantly enriched for genes associated with cytotoxic immune responses, with significant upregulation of key cytotoxic factors granzyme B (GzmB) and perforin (PFP), indicating that the cytotoxic function of these cells was activated at the transcriptional level. Subsequently, protein expression analysis revealed that the secretion of GzmB and PFP was increased in Mtb-HAg-activated γδ T cells. Meanwhile, intracellular Mtb growth inhibition assays demonstrated that activated γδ T cells lysed infected target cells and suppressed intracellular Mtb proliferation. We further found that BTN3A1 blockade significantly reduced the expression of CD69 and CD107a in γδ T cells, decreased the secretion of GzmB and PFP, and diminished the killing efficiency of γδ T cells against Mtb-infected macrophages.

**Conclusion:**

Our findings demonstrated that Mtb-HAg enhances the cytolytic activity of γδ T cells and inhibits intracellular Mtb growth, with BTN3A1 playing a regulatory role in these processes.

## 1 Introduction

Tuberculosis (TB), caused by Mycobacterium tuberculosis (Mtb), is a persistent global public health concern. Despite years of research, the disease persists, and Mtb has continued to evolve under selective pressure from long periods of drug treatment, leading to the emergence of multidrug-resistant TB (MDR-TB) and extensively drug-resistant TB (1). According to the World Health Organization (WHO), 10.8 million new cases of TB were reported globally in 2024, resulting in 1.25 million deaths, with approximately 400,000 new cases of MDR-TB being reported. These figures suggest that TB has likely regained its status as the world’s leading cause of death from a single infectious agent (2, 3). Given that the global burden of TB remains high and the limitations of traditional anti-TB drugs, adjuvant immunotherapy, particularly the use of unconventional T cells, has become a major focus of research in TB therapy over recent years (4–6).

Following Mtb invasion, the host immune system recognizes relevant TB antigens, leading to the activation of both innate and adaptive immune responses to clear the pathogen and provide protection against TB (7, 8). γδ T cells are a specialized subset of innate immune cells that display non-classical antigen recognition independently of the peptide-major histocompatibility complex (p-MHC) recognition paradigm. Instead, they exhibit broad specificity for non-peptide antigens such as phosphoantigens (pAgs), enabling direct antigen recognition and binding. This allows γδ T cells to serve as the first line of defense against infection and to connect with adaptive immunity for sustained protective effects (9). Notably, Mtb heat-resistant antigen (Mtb-HAg), a peptide antigen released into the supernatant of M. tuberculosis strain H37Ra after high-temperature and high-pressure treatment, has been shown to activate γδ T cells, specifically expand this subset, and enhance the secretion of cytokines, including interferon-gamma (IFN-γ), tumor necrosis factor-alpha (TNF-α), and interleukin-17 (IL-17) (10–12). Studies have shown that these cytokines mediate the killing of Mtb cells and the formation of TB granulomas, thereby controlling Mtb growth and spread (13–15). Our previous research revealed that Mtb-HAg acts on peripheral blood mononuclear cells (PBMCs), causing the early expansion of anti-TB immune cells and promoting the secretion of TB-protective cytokines. Multiple differentially expressed genes (DEGs) in these expanded activated cells were noted to be closely associated with Mtb infection and significantly enriched in pathways relevant to TB pathogenesis and progression (16, 17). While existing studies have demonstrated that the Mtb-HAg-activated γδ T cell populations produce elevated levels of key cytokines in anti-TB immunity, a comprehensive understanding of the antibacterial mechanisms of action of these cells is still lacking. Moreover, the immunomodulatory effects of Mtb-HAg and the molecular mechanisms underlying these effects are yet to be fully elucidated.

Studies have demonstrated that butyrophilins (BTN) are essential for γδ T cell activation. During pAg recognition, BTNs do not directly bind to the γδ T cell receptor (TCR). Instead, “presentation” involves interaction with the intracellular B30.2 domain of BTN3A1, which triggers γδ T cell activation (18, 19). Structural biology studies have revealed that the B30.2 domain of BTN3A1 directly binds pAgs and triggers transmembrane signaling via conformational changes, a process mediated by inside-out signaling (18, 20). The BTN3A1-pAg complex forms an interface capable of binding BTN2A1, with pAgs positioned centrally and adhering to BTN proteins with varying affinities. Subsequently, the exposed extracellular domains of this complex are recognized by γδ TCR through direct contact, enabling effective immune surveillance by γδ T cells (21, 22). Research has demonstrated that granzyme B (GzmB) expression in Vγ9Vδ2 T cells increases 20-fold within days of pAg stimulation (23). Furthermore, treatment with an agonistic anti-BTN3A1 monoclonal antibody (20.1 mAb) enhances γδ T cell toxicity against target cells (24), whereas BTN3A1 knockdown via short hairpin RNA (shRNA) significantly reduces the cytolytic activity and cytokine responses of γδ T cells against target cells treated with 20.1 mAb or soluble pAgs (25). These findings collectively indicate that BTN3A1 regulates the cytotoxic effector functions of activated γδ T cells. However, whether BTNs, and particularly BTN3A1, also participate in the amplification and enhancement of the cytotoxic functions of Mtb-HAg-activated γδ T cells remains unknown.

RNA sequencing (RNA-Seq) is a highly precise and sensitive technique that can be used to analyze differential gene expression in response to diverse stimuli. For instance, it has been used to reveal changes in gene expression during infection with virulent and non-virulent strains of Mtb (26–28). Recently, it has also been applied to the characterization of immune cells, such as macrophages and natural killer (NK) cells, during Mtb infection (29, 30). Additionally, single-cell RNA-Seq identified a Vδ2+ T-cell population with a mature cytotoxic phenotype. This subset uniquely exhibits high expression of killer cell lectin-like receptor (KLR) and killer cell immunoglobulin-like receptor (KIR) family genes. Notably, the molecular signatures of these cells showed significant correlations with the expression levels of classical cytotoxic proteins (e.g., perforin [PFP], GzmB), reflecting their intrinsic cytotoxic functions (31, 32).

In this study, we explored the biological functions and signaling pathways activated in γδ T cells following Mtb-HAg stimulation using transcriptome sequencing. Based on the results of transcriptomic enrichment, we extended our investigation of Mtb-HAg’s immunogenic activity beyond previous studies on anti-TB cytokine secretion to validate the cytotoxic functions and bactericidal capacity of Mtb-HAg-activated γδ T cells. This approach allowed us to further clarify the mechanism by which these cells suppress the survival of intracellular Mtb in macrophages and to confirm the enhanced immune resistance of these cells against Mtb infection. Additionally, we activated γδ T cell proliferation with Mtb-HAg, purified the activated cells, treated them with BTN3A1 antagonists, and co-cultured them with Mtb-infected macrophages to elucidate whether BTN3A1 influences Mtb-HAg-induced activation and the cytotoxic activity of γδ T cells.

## 2 Materials and methods

### 2.1 Blood donors and ethics statement

In this study, eight participants (5 males and 3 females) were recruited from Bengbu Medical University. All participants were healthy adults, aged 24 ± 3 years, had no recent history of infectious diseases or direct contact with TB, and showed normal chest X-rays on physical examination. Individuals with active TB, tumors, autoimmune diseases, and other conditions were excluded. All participants shared a common characteristic: they had received the BCG vaccine after birth. This research was reviewed and approved by the Human Ethics Committee of the Bengbu Medical University (approval no. BBMC-2022-68). Written informed consent was obtained from all participants.

### 2.2 Preparation of Mtb-HAg

Strain Mtb H37Ra was preserved by the Anhui Provincial Key Laboratory of Basic and Clinical Immunology of Chronic Diseases of Bengbu Medical University. Mtb-HAg was produced as previously specified (16, 33). Briefly, Mtb strain H37Ra was cultured for approximately 6 weeks, the mycobacteria in Sauton’s medium were centrifuged, and the bacterial pellet was washed three times with phosphate-buffered saline and once with ultrapure water. Twice the volume of ultrapure water was then added for further processing. The bacteria were autoclaved at 121°C for 20 min, and the supernatant was filtered through a 0.22-μm filter to acquire Mtb-HAg, which was subsequently used for stimulating γδ T cells.

### 2.3 Cell preparation

PBMCs were isolated from freshly collected EDTA-treated blood using density gradient centrifugation with human lymphocyte separation medium (TBD Science, Tianjin, China). The cells were aspirated, washed with RPMI 1640 medium (Gibco, USA), and cultured in RPMI 1640 medium supplemented with 1% penicillin-streptomycin-gentamicin mixture (Solarbio, China) and 10% newborn calf serum (Tianhang, China).

The PBMCs were cultured at an initial density of 1 × 10^6^ cells/mL in 24-well plates and incubated at 37°C in an atmosphere with 5% CO_2_. The cultures were then treated with rhIL-2 (PeproTech, USA) at 200 U/mL every 3 days, either in the presence or absence of 5 μg/mL Mtb-HAg. On day 14, the cells were collected for subsequent magnetic bead sorting of γδ T cells.

### 2.4 Sorting and identification of γδ T cells

First, the expanded cells were collected, and then thoroughly washed with autoMACS Running Buffer (Miltenyi, Germany). γδ T cells were then sorted from the PBMCs using a Human γ/δ T Cell Isolation Kit (Miltenyi, Germany). The cells were resuspended in 40 µL of autoMACS Running Buffer, followed by the addition of 10 µL of TCRγ/δ Hapten antibody. After incubation at 4°C in the dark for 15 min, 30 μL of autoMACS Running Buffer and 20 μL of anti-Hapten FITC microbeads were added, and the samples were thoroughly mixed and incubated at 4°C in the dark for 15 min. The magnetic separation column was then mounted onto a magnetic sorting frame and pretreated with 2 mL of autoMACS Running Buffer. The cell suspension was then loaded onto the column, and the target cells were isolated for use in subsequent experiments.

Cells were analyzed using a Cytek DxP Athena flow cytometer, both before and after purification. Cells were sorted based on their forward- and side-scatter properties. FlowJo CE was used for data collection, and FlowJo Analysis Software was employed for data analysis.

### 2.5 RNA-Seq analysis

DESeq was employed to identify DEGs between the IL-2 (control) and Mtb-HAg treatment groups, followed by principal component analysis (PCA) to evaluate DEG distribution patterns. The DEG screening criteria were set at P < 0.05 and |log2FoldChange| > 1. The “ggplot2” and “pheatmap” R packages were used to generate DEG volcano plots and heatmaps, respectively. To identify key enriched pathways and biological processes associated with the DEGs, Kyoto Encyclopedia of Genes and Genomes (KEGG; http://www.kegg.jp/) and Gene Ontology (GO; http://geneontology.org/) functional enrichment analysis was subsequently conducted using the R package “clusterProfiler”, with the significance threshold set at *P* < 0.05. Additionally, Gene Set Enrichment Analysis (GSEA) was conducted based on MSigDB (https://www.gsea-msigdb.org/gsea/msigdb) gene sets to explore functional differences and related pathways between the control and treatment groups, employing the criteria of |NES|>1, *P* < 0.05, false-discovery rate (FDR) <0.25, and q-value <0.05. A list of immune-related genes was downloaded from the ImmPort database (https://immport.org/) to screen for genes that overlapped with DEGs in the Mtb-HAg treatment group. PPI networks were established for these differentially expressed immune genes (DEIGs) using the STRING (version 12.0) online database (https://string-db.org/). The networks were then imported into Cytoscape (version 3.7.2) (https://cytoscape.org/) using the MCODE plug-in to identify key modules and elucidate the interaction relationships among the DEIGs. Finally, the key node genes in the DEIG PPI networks were identified using cytoHubba.

### 2.6 Analysis of the cell activation state

The anti-BTN3A1 mAb (clone 103.2) is an antagonistic antibody. It does not directly prevent the conformational change of the B30.2 domain. The core of its mechanism lies in binding to the extracellular Ig-V domain of the BTN3A, which hinders the recognition of the activated BTN3A state by the γδ TCR, stabilizes the extracellular domain in its native “V-shaped” dimers, and “locks” it in an inactive state, thereby inhibiting its activation (25, 34). Here, PBMCs freshly isolated from peripheral blood were seeded in 48-well plates (5 × 10^5^ cells/well) and divided into a control group, a stimulation group, a blocking group. Control group: untreated cells. Stimulation group: cells treated with Mtb-HAg (5 μg/mL). Blocking group: cells pre-treated with anti-BTN3A 103.2 mAb (10 μg/mL; Sino Biological, China) for 3 h, followed by addition of Mtb-HAg (5 μg/mL). After culturing for 24 h, the cells were collected and stained with FITC-labeled anti-human CD3 antibody, APC-labeled anti-human TCRγ/δ antibody, and PE-labeled anti-human CD69 antibody (all from Biolegend, USA), and analyzed on the Cytek DxP Athena flow cytometer. For BTN3A1 blockade, cells were pre-treated with the anti-BTN3A1 mAb (clone 103.2). Control cells were incubated in parallel under identical conditions without antibody addition. We note that an isotype control antibody was not used in this study; future experiments will incorporate this control.

### 2.7 Cytotoxicity analysis

γδ T cells obtained from expanded PBMCs from healthy volunteers were cultured and purified. Except for control wells, Mtb-HAg (5 μg/mL) was added to each well, with or without anti-BTN3A1 103.2 mAb (10 μg/mL). A subset of these cells was incubated with PE/Dazzle 594-labeled anti-CD107a antibody (Biolegend) and brefeldin A (BFA) (GolgiPlug, BD) for 4 h. The cells were then fixed in FluoroFix Buffer (Biolegend) and analyzed on the DxP Athena flow cytometer (Cytek, USA), with CD107a serving as a marker for the degranulation of cytotoxic molecules. Flow data were analyzed using FlowJo. Mtb-H37Ra-infected THP-1 cells—preserved by the Anhui Provincial Key Laboratory of Basic and Clinical Immunology of Chronic Diseases of Bengbu Medical University—were prepared as target cells at a multiplicity of infection (MOI) of 10, and any extracellular Mtb was washed away. Then, partially purified γδ T cells were co-cultured at 37°C with the target cells at an effector-to-target (E: T) ratio of 10:1. For the assessment of PFP/GzmB release, γδ T cells were harvested after 6 h of co-culture, repeatedly freeze-thawed with liquid nitrogen, and the resulting supernatant was collected. The Human Granzyme B Precoated ELISA Kit (Dakewe, China) and the Human Perforin Precoated ELISA Kit (CUSABIO, China) were used for the quantitative analysis of degranulation.

### 2.8 Intracellular mycobacterial growth inhibition assay

THP-1 cells were inoculated in 96-well plates at a density of 5 × 10^4^ cells/well with 100 ng/mL PMA and cultured for 24 h. Subsequently, the culture medium was removed, the adherent cells were washed twice with PBS and infected with Mtb-H37Ra in a complete medium without antibiotics at a MOI of 10. After 4 h of infection, the cells were washed with PBS. Meanwhile, γδ T cells were purified using a MACS separation column (Miltenyi Biotech) in accordance with the manufacturer’s protocol and the procedures described in Section 2.3 and were used as effector cells. Next, 5 μg/mL Mtb-HAg was added to each well, with or without the 10 μg/mL anti-BTN3A1 103.2 mAb. The target cells (5 × 10^4^ cells/well) were cultured with either the medium or the purified effector cells (5 × 10^5^ cells/well) in 96-well plates at an E:T ratio of 10:1 for 3 days. Intracellular bacteria were estimated by colony-forming unit (CFU) counting after cell lysis. Serial 10-fold dilutions of the cell lysates were plated on mycobacterial solid medium (Gene-Optimal, China) for quantitative culture. After incubation at 37°C for 5 weeks, the number of CFUs was calculated.

### 2.9 Statistical analysis

The criteria for statistical significance in the gene expression analysis are described in the corresponding individual methods sections. The data were analyzed using GraphPad Prism 8.0 software (GraphPad) within a completely randomized and controlled experimental design. The t-test was used to determine differences between two groups, while ANOVA was used to assess differences among three or more groups. For all analyses, *P* < 0.05 was used as the criterion for statistical significance.

## 3 Results

### 3.1 Identification of Mtb-HAg and purified γδ T cells

**Figure 1:**
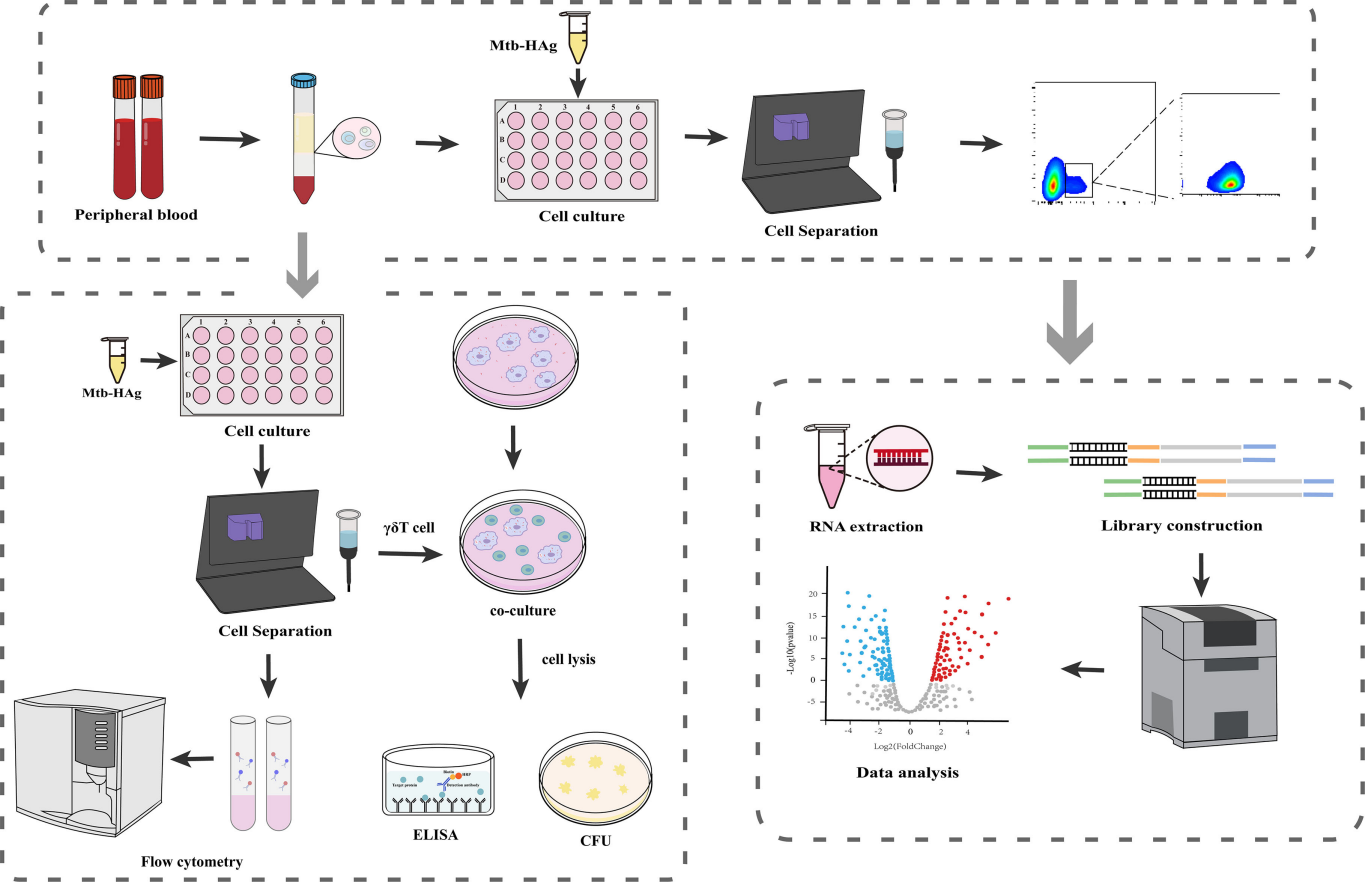
Workflow of transcriptome sequencing and analysis of Mtb-HAg regulation of γδ T cell function.

The experimental process is shown in **Figure 1**. Mtb-HAg was separated by 12.5% SDS-PAGE. The results showed that the components of Mtb-HAg were distributed in the range of 10–15 kDa, 25–35 kDa, 50 kDa, and 70 kDa (**Supplementary Figure 1A**). Stimulation of γδ T cells with Mtb-HAg confirmed that the extracted antigen effectively promoted their proliferation (**Supplementary Figures 1B, C**). To investigate gene expression in γδ T cells stimulated with Mtb-HAg and understand the mechanism underlying the response of the innate immune system to this stimulation, γδ T cells were sorted using magnetic beads, and the separation efficiency was assessed by flow cytometry. In all groups, the purity of γδ T cells was >95% (**Supplementary Figure 2**), thereby meeting the requirements for subsequent analyses.

### 3.2 Transcriptome profile and differentially expressed mRNAs

To further investigate the mechanism involved in the immunomodulatory effect of Mtb-HAg on γδ T cells, we performed transcriptome analysis using RNA-Seq. Raw reads were processed to remove low-quality reads and adapters, yielding a total of 1.25 billion clean reads. For each sample, the clean reads accounted for over 90% of the raw reads, with the Q30 exceeding 91%. Mapping to the human reference genome GRCh38 v38.82 yielded alignment rates ranging from 94% to 96%, meeting the criteria for subsequent analysis (**Supplementary Table 1**).

**Figure 2:**
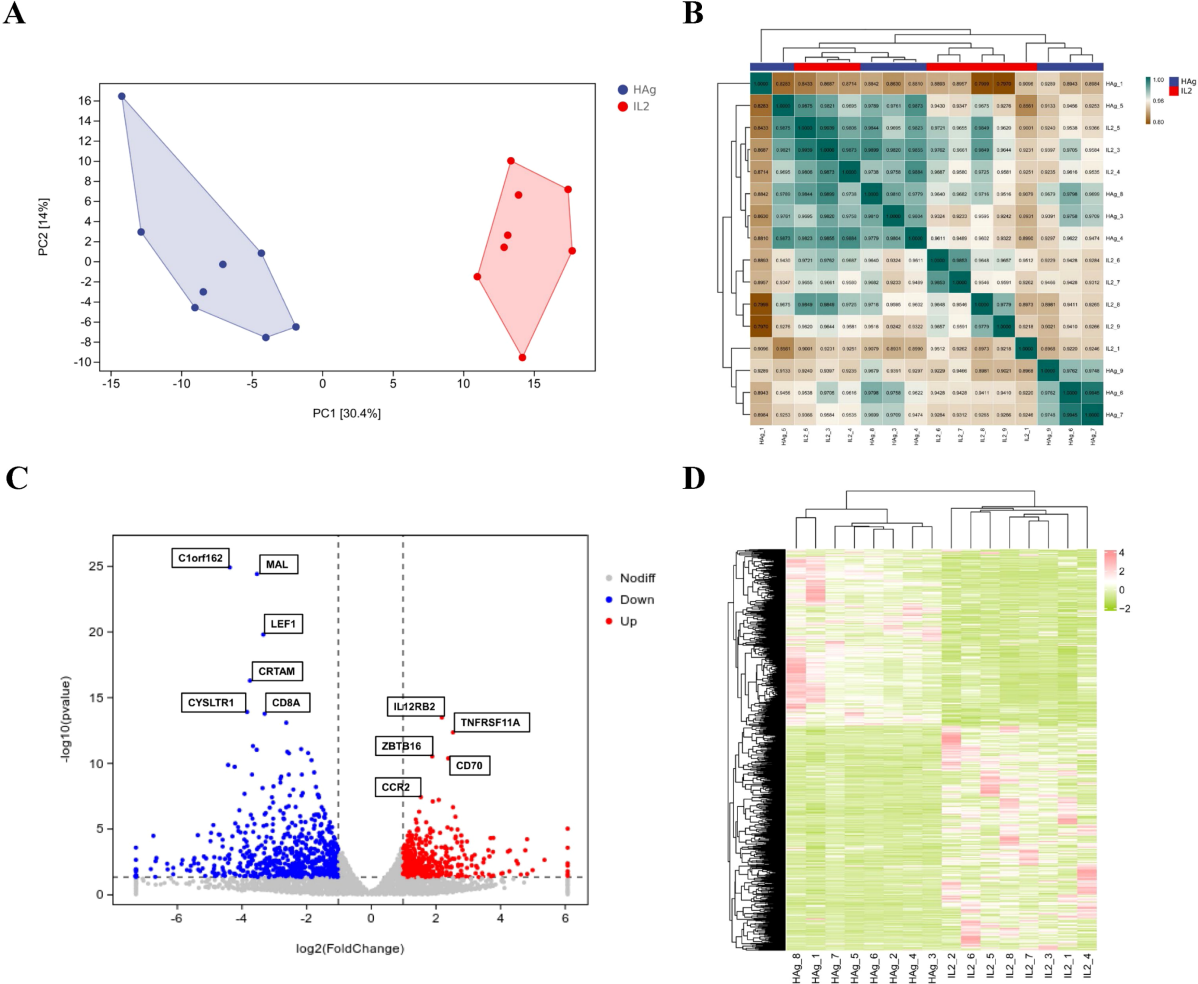
Transcriptome profile and identification of differentially expressed genes. **(A)** Principal Component Analysis (PCA) of transcriptome data for the Mtb-HAg and control groups. **(B)** Pearson’s correlation analysis of the transcriptome data from the two treatment groups. **(C)** Volcano plot of the differentially expressed genes between the Mtb-HAg and control groups. Red dots represent upregulated genes, blue dots represent downregulated genes, and gray dots indicate non–differentially expressed genes (*P* < 0.05 and |log2FoldChange| > 1). **(D)** Cluster analysis of transcriptome data from the Mtb-HAg and control groups. (n = 8 per group).

First, we used PCA and the Pearson correlation coefficient to evaluate the differences and correlations among the experimental samples. PCA plots showed that the Mtb-HAg groups were significantly separated from the control group, indicative of distinct transcriptional profiles (**Figure 2A**). Correlation matrix plots, which show correlations of gene expression levels between groups and the within-group reproducibility of changes, revealed that the variation between replicates was small, whereas the correlation between expression levels was high, thus demonstrating the relative reliability of our data (**Figure 2B**). Secondly, a total of 1,142 DEGs were identified, including 526 that were upregulated and 616 that were downregulated (**Figure 2C**). In addition, we performed a hierarchical clustering analysis of the DEGs, and the results were visualized as a heatmap. We observed that the expression profiles of γδ T cells differed significantly among the three experimental conditions (**Figure 2D**).

### 3.3 GO function analysis, KEGG pathway enrichment analysis, and GSEA

**Figure 3:**
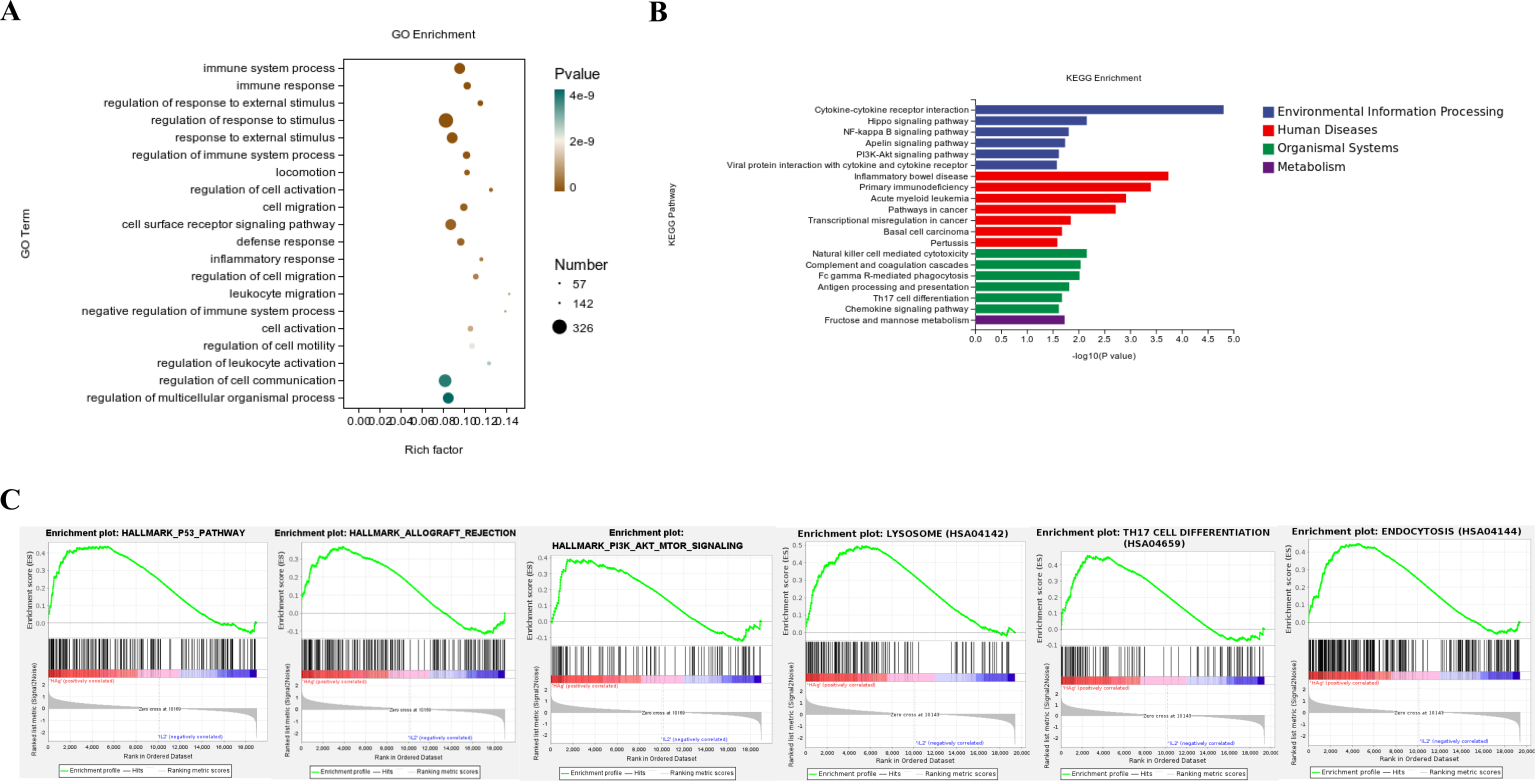
GO, KEGG, and GSEA of the differentially expressed genes. **(A)** GO term enrichment analysis of the differentially expressed genes between the Mtb-HAg and control groups. **(B)** KEGG pathway enrichment analysis of the differentially expressed genes between the Mtb-HAg and control groups. **(C)** GSEA of the expressed genes between the Mtb-HAg and control groups.

To investigate the biological functions and enriched pathways associated with the identified DEGs, we performed GO annotation and KEGG enrichment analysis of the DEGs between the Mtb-HAg and control groups. The results showed that the DEGs in the Mtb-HAg group were mainly enriched in immune system processes and regulation of immune system processes, immune response, regulation of response to external stimulus, and cell activation regulation (**Figure 3A**). Meanwhile, KEGG pathway analysis showed that the DEGs in the Mtb-HAg group were enriched in cytokines and cytokine receptor signaling pathways, the Hippo signaling pathway, and the nuclear factor-kappa B signaling pathway. These DEGs were also involved in NK cell-mediated cytotoxicity, antigen processing and presentation, and Th17 cell differentiation, among other pathways (**Figure 3B**).

GSEA was used to analyze all the expressed genes within each group to more broadly identify the heterogeneous changes occurring in γδ T cells following antigen recognition. The results showed that the Mtb-HAg group showed enrichment of 41 hallmark gene sets, including those associated with the p53 pathway, the PI3K-AKT-mTOR signaling pathway, hypoxia, and apoptosis. GSEA-based KEGG-enrichment plots further showed enrichment in pathways linked to the lysosome, Th17 cell differentiation, and endocytosis in the Mtb-HAg group (**Figure 3C**).

### 3.4 PPI network construction and module analysis

**Figure 4:**
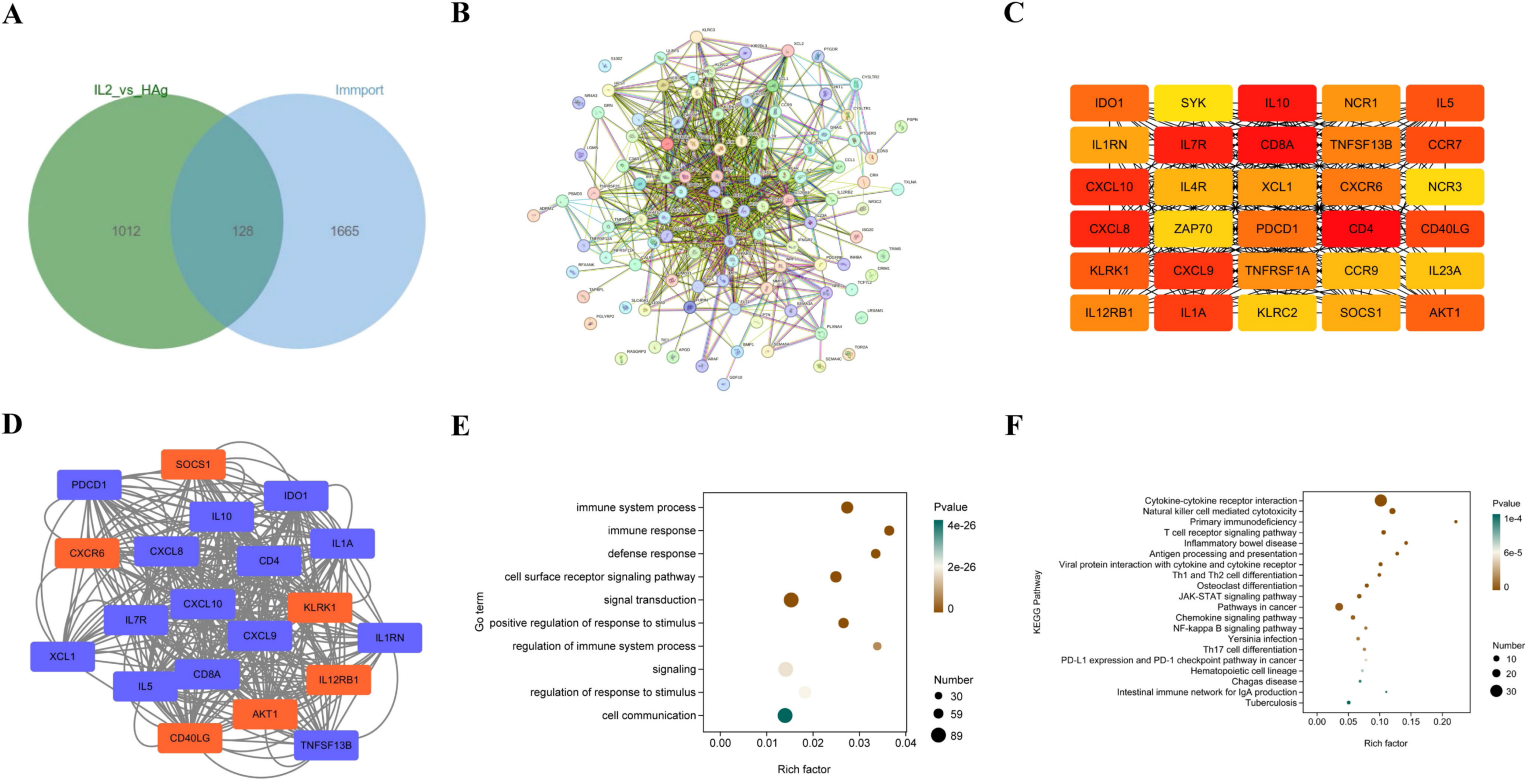
Analysis of differentially expressed immune-related genes (DEIGs). **(A)** Venn diagram DEIGs in the Mtb-HAg group. **(B)** Protein-protein interaction (PPI) network for the DEIGs in the Mtb-HAg group. **(C)** The top 30 hub DEIGs in the Mtb-HAg group. **(D)** Key modules for the DEIGs in the Mtb-HAg group. **(E)** GO enrichment analysis of the DEIGs in the Mtb-HAg group. **(F)** KEGG pathway enrichment analysis of the DEIGs in the Mtb-HAg group.

Next, we identified DEIGs in the Mtb-HAg group by cross-referencing the list of immune-related genes obtained from the Immport database with the DEGs in the Mtb-HAg group (**Figure 4A**). To further characterize the cellular regulatory mechanisms and functions of the associated proteins, we performed a PPI network analysis of the DEIGs (**Figure 4B**). Hub genes that may play important biological roles in the interaction networks were screened using the Cytoscape plug-in Cytohubba, with the top 30 hub genes being selected for the DEIG interaction network in the Mtb-HAg group (**Figure 4C**).

From this network, four modules were extracted, with the main module having a k-core score of 17.263 (**Figure 4D**). Further functional analysis of these genes revealed that they were involved in biological processes such as immune system processes, immune response, and defense response, and were enriched in the cytokine-cytokine receptor signaling pathway, JAK-STAT signaling pathway, NK cell-mediated cytotoxicity, T cell receptor signaling pathway, and antigen processing and presentation (**Figure 4E**). KEGG analysis of the main modules showed that they were mainly related to pathways associated with cytokine-cytokine receptor signaling, JAK-STAT signaling, T cell receptor signaling, NK cell-mediated cytotoxicity, and Th1/Th2/Th17 cell differentiation (**Figure 4F**).

### 3.5 Visualization of cytotoxicity-related pathway enrichment and the associated gene expression in Mtb-HAg-activated γδ T cells

**Figure 5:**
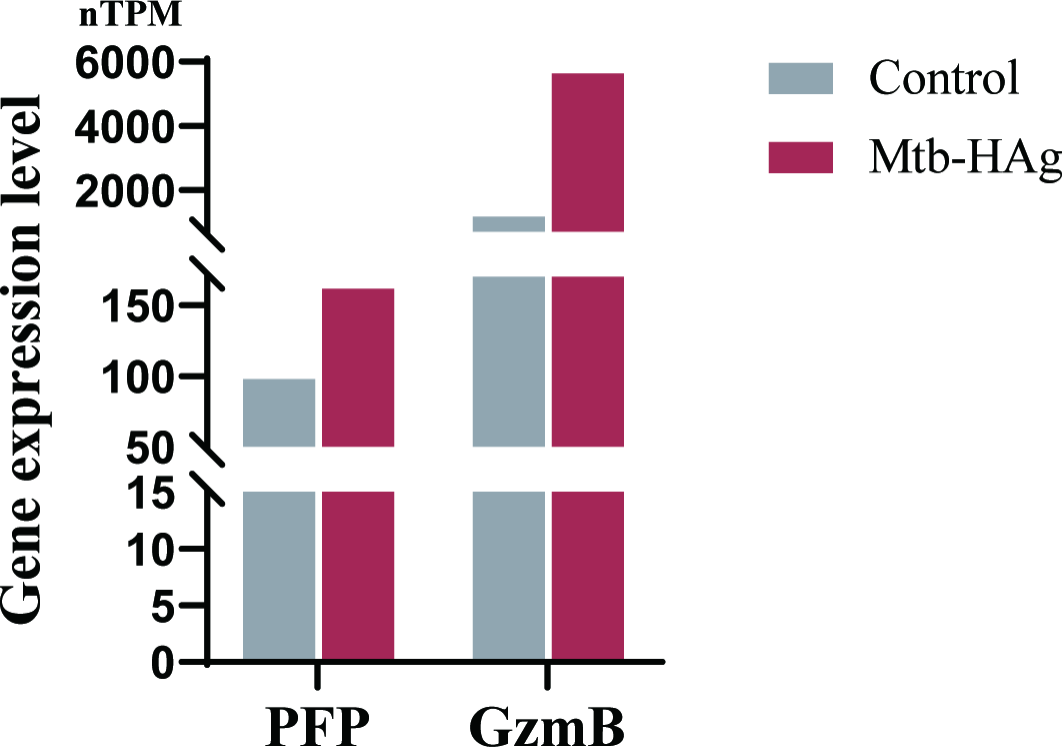
Expression levels of the cytotoxic molecule genes granzyme B (GzmB) and perforin (PFP).

RNA-Seq analysis indicated that the DEGs in the Mtb-HAg group exhibited significant enrichment in cytotoxicity-related pathways. It has been shown that BCG vaccination markedly enhances the immune effector functions of peripheral blood γδ T cells, characterized by significantly upregulated expression levels of the pro-inflammatory cytokines IFN-γ and TNF-α, accompanied by substantial increases in PFP secretion and the proportion of GzmB+ γδ T cell subsets (35). Our group has also previously demonstrated that Mtb-HAg can augment IFN-γ and TNF-α secretion by γδ T cells (17). The RNA-Seq results in the current study revealed the existence of a strong correlation between cytotoxicity and Mtb-HAg-activated γδ T cells, reflected in the upregulation of the expression of the cytotoxic factor-encoding genes GZMB and PFP (**Figure 5**).

During the activation of γδ T cells, the intracellular domain of BTN3A1, B30.2, is a key site for antigen binding and plays a crucial role in triggering γδ T cell activation. We have previously also demonstrated that BTN3A1 promotes Mtb-HAg-induced activation and proliferation of γδ T cells, and also modulates the release of TNF-α and IFN-γ by these cells (36). Notably, the deletion of the BTN3A1 B30.2 domain has been shown to impair pAg-induced degranulation of γδ T cells (37). Therefore, in this study, we sought to determine whether BTN3A1 influences the Mtb-HAg-induced degranulation activity of γδ T cells. For this, we activated γδ T cells with Mtb-HAg, inhibited BTN3A1 expression through the addition of an anti-BTN3A monoclonal antibody (antagonist) and evaluated both the release of cytotoxic factors and the intracellular Mtb-killing function of the γδ T cells.

### 3.6 Mtb-HAg can significantly enhance the secretion of GzmB and PFP by γδ T cells, and inhibit Mtb intracellular growth

**Figure 6:**
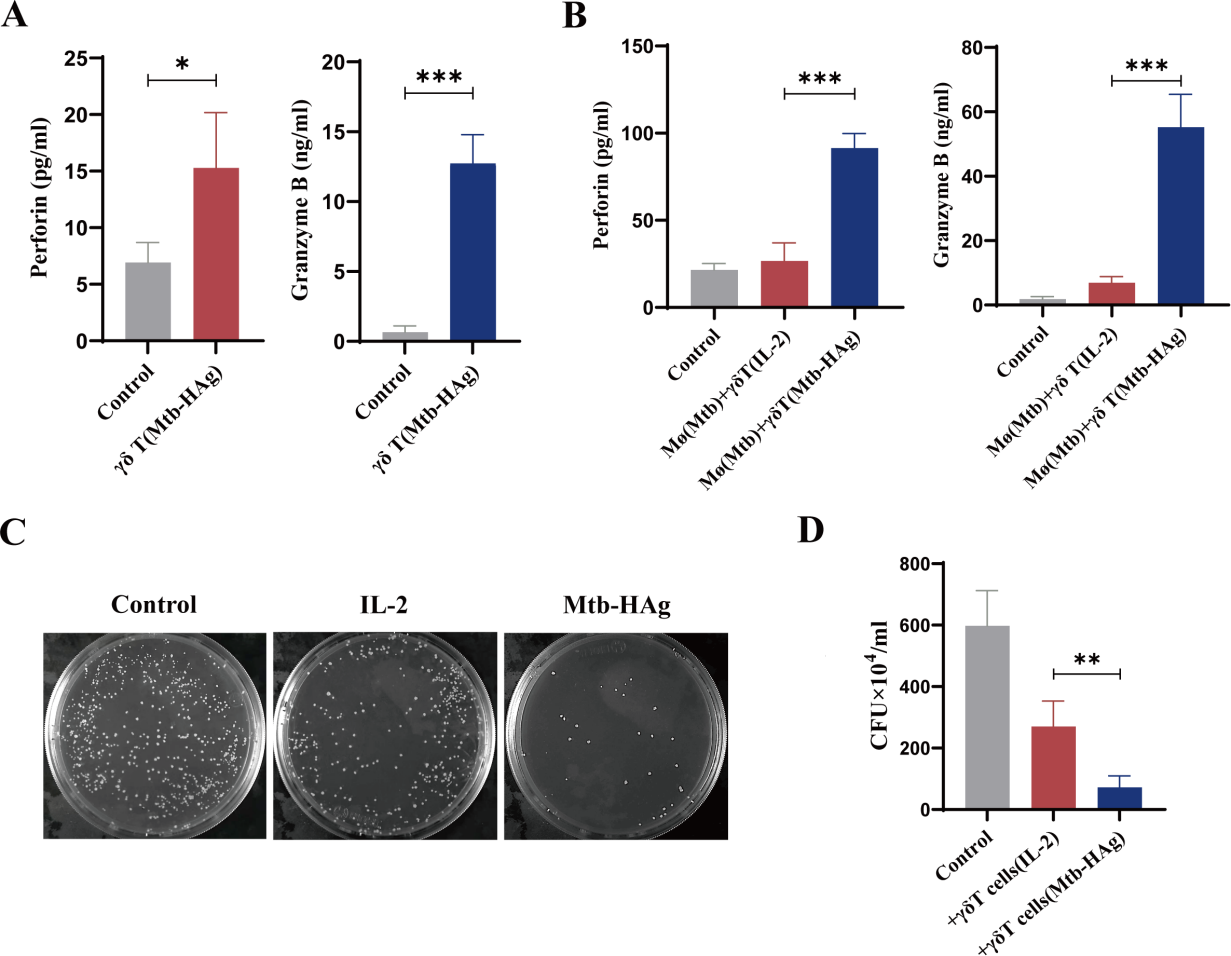
Mtb-HAg amplified γδ T cell lytic activity and inhibited the growth of intracellular mycobacteria. **(A)** Mtb-HAg promoted the secretion of granzyme B and perforin by γδ T cells. **(B)** The release of perforin and granzyme B from Mtb-HAg activated γδ T cells in a co-culture system, as detected by ELISA. **(C)** γδ T cells activated by Mtb-HAg inhibited Mtb growth in THP-1-derived macrophages. **(D)** Statistics relating to the inhibition of intracellular bacterial growth. (*P < 0.05, **P < 0.01, ***P < 0.001).

Because mRNA levels reflect transcriptional activity, they can only indicate the potential capacity of Mtb-HAg-activated γδ T cells to secrete GzmB and PFP and cannot directly confirm protein secretion. Therefore, we first used ELISAs to confirm that GzmB and PFP secretion levels indeed change in γδ T cells under Mtb-HAg stimulation. The results demonstrated that Mtb-HAg-activated γδ T cells exhibited significantly increased release of GzmB and PFP compared to control cells (**Figure 6A**), which supported our subsequent in-depth investigations.

Given that the granzyme-perforin pathway in cytotoxic immune responses predominantly relies on direct cell-to-cell contact to exert its potent immune activity, we established a co-culture system comprising effector cells and infected target cells to investigate the involvement of granular molecules (GzmB and PFP) of cytolytic T lymphocytes (CTLs) in eliminating intracellular pathogens under direct cellular contact, as well as their roles in infected cell lysis and bacterial growth. We found that activated γδ T cells from the Mtb-HAg-treated group produced more GzmB and PFP than those from the untreated control group (**Figure 6B**).

Subsequently, we tested the effect of these amplified cells on the inhibition of Mtb growth in macrophages. As previously mentioned, THP-1-derived macrophages were infected with Mtb and either cultured alone or co-cultured with Mtb-HAg-activated γδ T cells. After 6, 24, 48, and 72 h, we observed that activated γδ T cells could rapidly recognize infected macrophages and later lyse them (**Supplementary Figure 3**). In addition, we performed a lytic culture of the co-culture products and found that γδ T effector cells purified from Mtb-HAg treatment cultures inhibited Mtb growth in target cells to a significantly greater extent than control medium alone (**Figure 6C**). This suggested that Mtb-HAg-activated γδ T effector cells exert potent cytotoxic effects, leading to the suppression of intracellular Mtb growth via the granzyme-perforin pathway.

### 3.7 BTN3A1 antagonists inhibited the cytotoxicity-enhancing effect of Mtb-HAg on γδ T cells

Based on the unique antigen recognition pattern of γδ T cells, the critical role of the intracellular B30.2 domain of BTN3A1 in initiating γδ T cell activation, and the impact of this molecular structure on the cytotoxic function of γδ T cells, we hypothesized that the effects induced by Mtb-HAg may share similarities with those promoted by pAgs. To explore whether Mtb-HAg-induced γδ T cell activation and cytotoxicity are regulated by BTN3A1, we next applied BTN3A antagonists. CD107a is predominantly localized on the surfaces of lysosomes and cytotoxic granules. The surface expression level of CD107a directly reflects cellular degranulation activity and serves as a key dynamic indicator for evaluating cytotoxic functions (38). The flow cytometry gating strategy is shown in **Supplementary Figure 4**.

**Figure 7:**
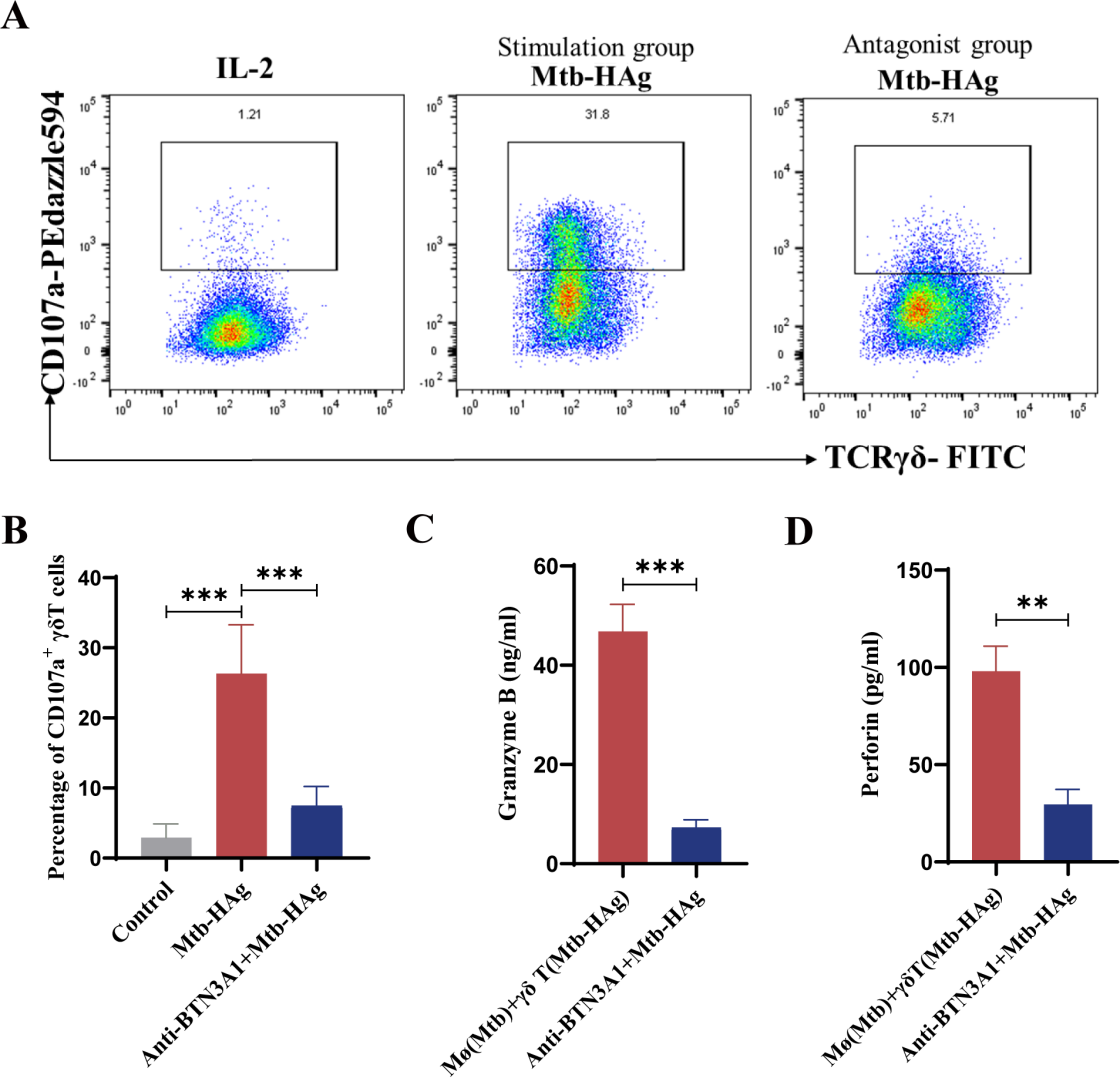
The cytotoxic effect of Mtb-HAg-activated γδ T cells was inhibited by treatment with a BTN3A1 antagonist. **(A)** Flow cytometric analysis of the expression of CD107a in Mtb-HAg-stimulated γδ T cells. **(B)** Statistics relating to the CD107a+ γδ T cell positivity rate. **(C)** ELISA for the detection of perforin release from γδ T cells activated by Mtb-HAg following treatment with the BTN3A1 antagonist. **(D)** ELISA for the detection of granzyme B release from γδ T cells activated by Mtb-HAg following treatment with the BTN3A1 antagonist. (**P < 0.01, ***P < 0.001).

Flow cytometry was used to assess changes in the expression levels of the cytotoxic factor CD107a under Mtb-HAg stimulation. A comparative analysis of CD107a+ γδ T cell subsets revealed that the proportion of this subtype was significantly higher in the Mtb-HAg group (26.33 ± 6.95%) than in the control group (2.92 ± 1.96%). The group undergoing stimulation also displayed a higher percentage of CD107a-positive cells (**Figure 7A**). Having confirmed that Mtb-HAg treatment indeed led to changes in CD107a expression, BTN3A1 antagonists were introduced to the cultures, and an effector-target cell co-culture system was established to validate the regulatory role of BTN3A1 in the killing process. The data demonstrated that, following BTN3A1 inhibitor treatment, Mtb-HAg-activated γδ T cells exhibited a reduction in CD107a expression and a diminished capacity for producing GzmB and PFP (**Figure 7**). The observed attenuation of cytotoxicity following BTN3A antagonist treatment can be at least partially attributed to the involvement of BTN3A1 in the functional modulation of Mtb-HAg-activated γδ T cells. Combined, these observations suggested that targeting this molecule may enhance the bactericidal efficacy of Mtb-HAg-activated γδ T cells.

### 3.8 BTN3A1 antagonism suppresses the significant Mtb-HAg-mediated upregulation of CD69 expression in γδ T cells

**Figure 8:**
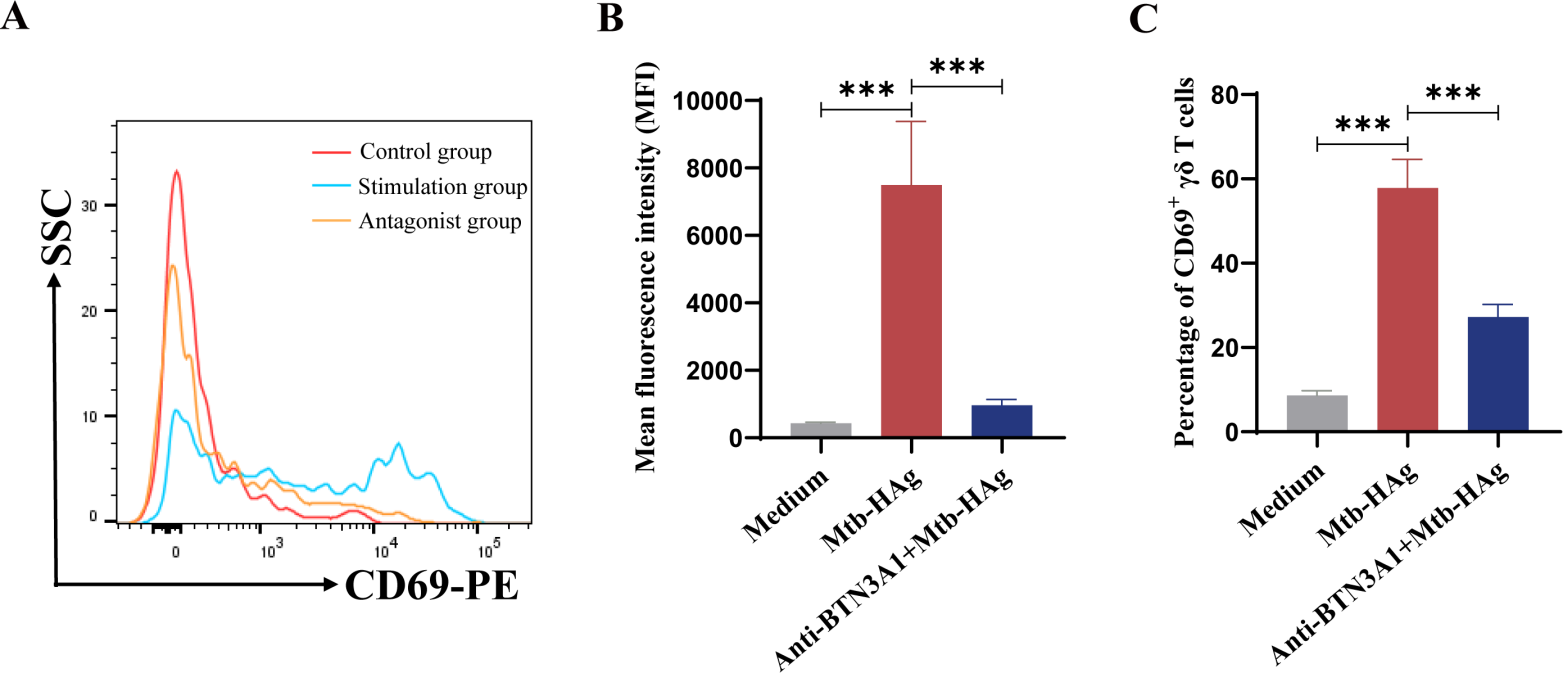
Flow cytometric analysis of Mtb-HAg-induced CD69 expression on γδ T cells. **(A)** The expression of CD69 on γδ T cells induced by Mtb-HAg, as determined by flow cytometry. **(B)** Statistics relating to the mean fluorescence intensity of CD69+ γδ T cells. **(C)** Statistics relating to the CD69+ γδ T cell positivity rate (***P < 0.001).

Before activated γδ T cells transform into cytotoxic effector cells, the changes induced by activation stimuli are first reflected in the surface expression of activation markers. CD69 is an early marker of T-cell activation that can be directly evaluated by flow cytometry. To further investigate the cytotoxic effect of activated γδ T cells and whether BTN3A1 is involved in this process, we next evaluated the influence of Mtb-HAg on γδ T cells using flow cytometry. The samples were collected 24 h after Mtb-HAg addition. Flow cytometry results indicated that the mean fluorescence intensity (MFI) of CD69 in Mtb-HAg-stimulated γδ T cells was 7499 ± 1877, which was significantly higher than the 429.7 ± 33.50 observed in control cells (**Figure 8B**). Similarly, the percentage of CD69-positive cells was significantly higher in the Mtb-HAg stimulation group (57.80 ± 6.82%) than in the control group (8.66 ± 1.11%) (**Figure 8C**). However, when anti-BTN3A1 103.2 mAb was introduced to Mtb-HAg-treated γδ T cell cultures, the MFI of CD69 dropped to 961 ± 180.1. Additionally, the percentage of CD69-positive cells was 27.23 ± 2.97%, substantially lower than that in the Mtb-HAg stimulation group, as were the expression levels of activation markers in γδ T cell subsets (**Figure 8**). These results further supported that BTN3A1 is involved in the regulation of Mtb-HAg-activated γδ T cells.

### 3.9 Antagonizing BTN3A1 significantly inhibited the ability of Mtb-HAg-activated γδ T cells to kill intracellular Mtb

**Figure 9:**
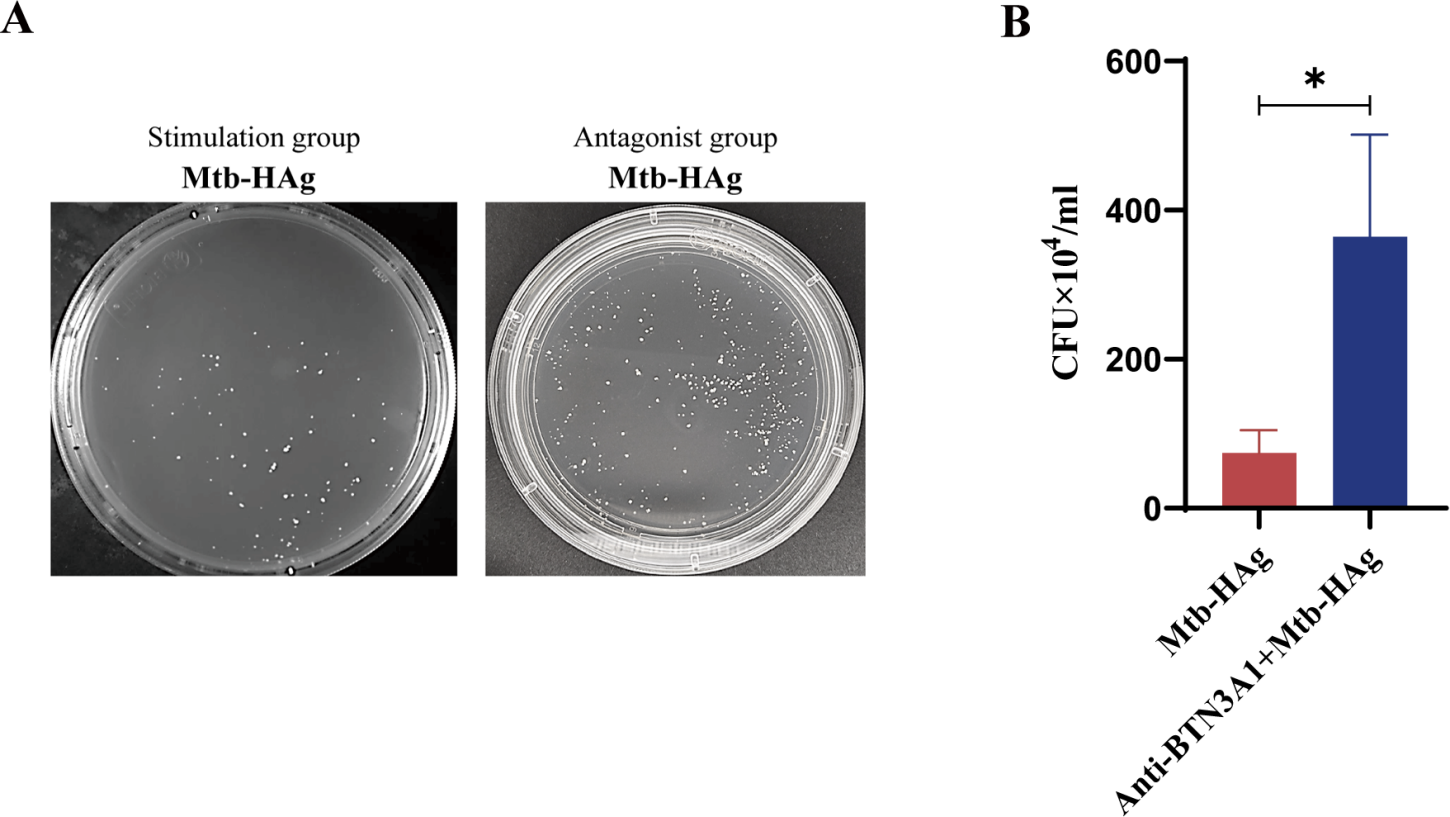
A BTN3A1 antagonist impaired intracellular Mtb killing by Mtb-HAg-activated γδ T cells. **(A)** The effect of the BTN3A1 antagonist on the ability of γδ T cells to kill intracellular Mtb. **(B)** Statistics relating to the inhibition of intracellular bacterial growth (*P < 0.05).

Given that Mtb-HAg-amplified γδ T cells exhibit enhanced release of cytotoxic factors that inhibit the survival of intracellular Mtb, and that BTN3A1 partially modulates the production of these factors, it is important to determine whether the capacity of these amplified cells to directly restrict the growth of intracellular Mtb is attenuated under BTN3A1 antagonism. Here, we evaluated the effect of an antagonistic anti-BTN3A 103.2 monoclonal antibody on the efficacy of γδ T cell-mediated killing of intracellular Mtb. Compared to the control and stimulation groups, Mtb-HAg-activated γδ T effector cells under BTN3A1 antagonism demonstrated a significant reduction in their capacity to inhibit Mtb growth within target cells (**Figure 9**).

## 4 Discussion

The continuous mutation of Mtb has established it as an untreatable pathogen globally. A crucial step toward effectively managing TB is to understand the mechanisms governing the interaction between the host immune system and the pathogen (39, 40). In recent years, γδ T cells have gained prominence, with potential applications involving infection control—particularly for TB—in addition to their well-known role in cancer treatment (41, 42). Global TCR sequencing has been used to investigate the role of these cells in the human lung during TB. The results showed that the number of γδ T cells was markedly increased in the lung tissues and matched blood of individuals with TB relative to that in healthy individuals and patients with other lower respiratory tract infections, and the γδ T cell population was characterized by highly localized clonal expansions (43). The reported amplification of γδ T cells in household contacts of TB patients (44) supports that these cells play a potential role in the host response to TB. However, active TB has also been linked to the depletion of circulating γδ T cells (45), and γδ T cell levels have been reported to fluctuate to varying degrees in patients with different disease conditions. The loss and depletion of γδ T cells in the blood and lungs have been strongly associated with disease progression (46, 47). These findings suggest that γδ T cells may be involved in anti-infection immunity through specific recruitment to infection sites or the clonal expansion of tissue-resident γδ T cells. With the continued development of high-throughput sequencing technologies, highly specific gene expression profiles can now be generated. Human peripheral blood transcriptome analysis can help to identify subsets with essential roles in anti-TB immunity, which is crucial for furthering the understanding of the host immune response to Mtb (48). Therefore, in this study, we comprehensively analyzed the mRNA expression profiles of γδ T cells from control and Mtb-HAg groups using RNA-Seq. This allowed for the exploration of the effects of Mtb-HAg on the immune function of γδ T cells, as well as the associated signaling pathways and potential molecular mechanisms at the mRNA level.

In this study, all transcripts of γδ T cells in the Mtb-HAg treatment and control groups were comprehensively screened. Subsequently, GO, KEGG, and GSEA were performed on the identified DEGs. The GO terms were primarily associated with T-cell regulation and the response to external stimuli, suggesting that Mtb-HAg-treated γδ T cells possess immunoregulatory activity. Regarding KEGG pathways, the DEGs were predominantly enriched in cytokine-cytokine receptor signaling and among others, the NF-κB signaling pathway. The significant enrichment observed in the NK cell-mediated cytotoxicity pathway within organismal systems was particularly noteworthy. Cell cytotoxicity is a critical immune effector mechanism. For instance, NK cells and CTLs eliminate pathogen-infected cells through the release of the contents of cytolytic granules (49). Activated γδ T cells have been shown to lyse Brucella-infected monocytes and inhibit the intracellular growth of pathogenic Brucella via the perforin-granzyme pathway (50). This mechanism also enables the elimination of intracellular protozoan parasites such as Trypanosoma cruzi, Toxoplasma gondii, and Leishmania major (51). In non-human primate (NHP) models, the *in vivo* administration of pAgs promotes γδ T cell expansion, leading to the production of anti-TB cytokines such as IFN-γ, perforin, and granulysin (GNLY). These activated γδ T cells migrate to Mtb-infected lung tissues, where they release perforin and granulysin. Granulysin-producing γδ T cells directly restrict intracellular Mtb growth through mycobactericidal activity, while perforin-mediated suppression further enhances the containment of bacterial replication. This dual mechanism significantly reduces the pulmonary bacterial load and lessens TB pathology in infected lung tissues (52).

GSEA also demonstrated that the enriched sets were highly correlated with lysosomes. Previous studies have shown that the granules secreted by CTLs, which contain perforin and granzymes and are released during specific interactions with target cells, are secretory lysosomes. The lysosomal membrane glycoproteins LAMP-1, LAMP-2, and CD63 are abundantly present on the granule-delimiting outer membrane. This membrane is incorporated into the plasma membrane of CTLs during a fatal hit (53), releasing soluble granzymes and PFP from the secretory lysosomes into the cytotoxic immunological synapse—the interface between the CTL and the target cell (54)—thereby exerting their immune effects. In addition to these enriched pathways, we also screened for additional DEIGs. Among the hub genes, KLRK1, encoding natural killer group 2 member D (NKG2D), was significantly upregulated. NKG2D functions as a cytotoxic receptor and participates in the positive regulation of NK cell-mediated cytotoxicity. In multiple murine studies, its engagement on γδ T cells has been demonstrated to induce degranulation, cytotoxicity, and, occasionally, cytokine production (55–57). The cytotoxicity of γδ T cells is γδ TCR-dependent and can be co-stimulated by NKG2D. This NKG2D interaction enhances the activation of γδ T cell cytotoxicity through the Vav1-phospholipase C-γ1 pathway (58). Furthermore, in patients infected with *M. tuberculosis*, the binding of UL-16-binding protein 1 to NKG2D on T cells contributes to the production of IL-23-dependent IL-17 (59). The results of our transcriptome sequencing, which indicated that a strong correlation exists between Mtb-HAg-activated γδ T cells and cytotoxicity, provided a basis for further experimental validation.

The rarity of γδ T cells in peripheral blood presents a challenge for their clinical application. To enhance the therapeutic potential of these cells, it is necessary to identify and develop more suitable antigens capable of promoting a strong immune response. Previous work involved the extraction, purification, and identification of Mtb-HAg and its components. This included protease treatment of Mtb-HAg, which reduced its bioactivity toward both resting and activated γδ T cells (10). Additionally, liquid chromatography-mass spectrometry (LC-MS) was employed to separate and characterize Mtb-HAg. Antigenic components screened from Mtb-HAg were further identified and functionally analyzed, confirming their effects on γδ T cells (17) and thus providing preliminary evidence for their immunomodulatory activity. It also demonstrated that Mtb-HAg is a polypeptide component and that the components functioning are essentially only proteins/peptides. Previous transcriptomic and quantitative real-time PCR analyses revealed that Mtb-HAg stimulation significantly upregulates the expression of genes such as IFNG and TNF (16), a finding consistent with flow cytometry results (17). Together, these data demonstrate that Mtb-HAg-activated γδ T cells contribute to anti-tuberculosis immunity via the secretion of antimicrobial cytokines. Building upon the observed changes in cytotoxic factors induced by Mtb-HAg, this study further systematically evaluated the cytotoxic immune activity of Mtb-HAg-induced γδ T cells by assessing cell activation levels, the expression levels of CD107a, GzmB, and PFP; and the effect on intracellular mycobacterial proliferation. The results showed that Mtb-HAg not only promotes the activation and expansion of γδ T cells but also enhances the release of the antibacterial substances GzmB and PFP by these cells, leading to strong cytolytic activity against infected cells. The primary function of PFP is to form transmembrane pores on the cell membranes of target cells, facilitating the diffusion of granzymes and other substances into their cytoplasm (60). Mouse knockout studies have shown that the deletion of perforin eliminates granule-dependent target cell death and provided evidence that it plays a key role in promoting granzyme-induced apoptosis (61, 62). Once GzmB enters cells, it can weaken the defenses of intracellular microbes against oxygen radicals, increasing their susceptibility to oxidative damage and promoting bacterial death, thereby inhibiting their growth (63). Studies on soluble factors produced by γδ T cells have established a significant correlation between granzyme production levels and the inhibition of intracellular Mtb growth (64). This inhibitory activity is compromised when the corresponding gene is knocked out (65). In NHP models of Mtb infection, γδ T cells have been observed within granulomatous tuberculous lesions in post-infection lung tissues, where they produce the cytotoxic granular molecule GzmB (66). These studies collectively indicate that the cytotoxic factors GzmB and PFP are instrumental in the immune response to TB infection.

In this study, γδ T cells stimulated with Mtb-HAg displayed significantly enhanced cytotoxic function. By measuring the secretion of cytotoxic granules, we found that the release of GzmB and PFP from γδ T cells was significantly increased in the experimental group compared with that in the controls. The mechanism involved in granule secretion in cytotoxic T lymphocytes (CTLs) is very rapid, with target cell lysis occurring within just a few minutes *in vitro* (67). We further observed that the expression of CD107a in activated γδ T cells was significantly higher in all treatment groups than in the control group. This indicates that the cytolytic activity of γδ T cells was substantially enhanced by Mtb-HAg, which is consistent with the observed increased secretion of cytotoxic granules and the consequent lysis of target cells. Moreover, this enhanced cytotoxic function of Mtb-HAg-activated γδ T cells was strongly correlated with the inhibition of Mtb growth in macrophages. Combined, these findings demonstrate that Mtb-HAg can inhibit the proliferation of intracellular pathogens by regulating γδ T cell cytotoxic function, thereby establishing its role in immune regulation.

In addition, studies have shown that chronic Mtb infection leads to the expansion of a unique subpopulation of γδ T cells with a “memory inflation” feature. These cells can produce a powerful cytotoxic response, and their clones are mycobacteria-specific but not pAg-reactive (68). This indirectly suggests that the immune function of γδ T cells during TB infection does not rely solely on pAgs. It has been demonstrated that γδ T cells isolated from BCG-vaccinated individuals can recognize BP3, a peptide derived from the oxidative stress response regulatory protein of Mycobacterium bovis BCG (69). Similarly, in animals infected with virulent M. bovis, γδ T cells proliferate and produce IFN-γ upon specific and direct contact with complex mycobacterial antigens such as PPD-B and the ESAT6:CFP10 protein complex (70). Furthermore, a study showed that using tumor-associated antigen phosphatidylinositol proteoglycan-3 (GPC3)-derived peptides as antigens can enhance the expansion efficiency of GPC3-specific CTLs and elicit GPC3-specific cytotoxicity. The adoptive transfer of effector cells expanded with these peptide antigens plus zoledronic acid in NOD/SCID mouse models significantly inhibited tumor growth, establishing an innovative strategy for enhancing antigen-specific immunotherapy (71). Based on these insights, we extracted a related peptide antigen directly from Mtb to stimulate γδ T cells, confirmed the specific immune response, and demonstrated the feasibility and effectiveness of our extracted antigen for the study and diagnosis of TB infection.

γδ T cells and αβ T cells exhibit significant differences in antigen recognition. A key discovery is that the activation of γδ T cells involves the substantial participation of BTN and BTN-like molecules (25). The B30.2 domain of the BTN3A1 molecule undergoes a conformational change upon binding to a pAg, and the γδ T-cell receptor (TCR) can recognize this change (20, 34, 72). This, in turn, enhances the killing effect of γδ T cells (73). Recent studies have shown that during Plasmodium falciparum infection, infected red blood cells (iRBCs) stained for BTN3A1 are recognized by γδ T cells. These cells form immune synapses and lyse iRBCs in a manner dependent on contact, pAg, BTN3A1, and degranulation, thereby killing the intracellular parasites (74). In a different study involving a human acute myeloid leukemia (AML) xenotransplantation mouse model, it was demonstrated that an anti-BTN3A 20.1 mAb enhanced the activity and killing effect mediated by Vγ9Vδ2 T cells. The simultaneous incubation of Vγ9Vδ2 T cells with the anti-BTN3A 20.1 mAb and AML blasts *in vitro* led to a further enhancement of cytotoxicity, which was abolished with the use of BTN3A antagonists (75). These findings highlight the potential of the BTN subfamily cooperative pathway as a therapeutic target for activating γδ T cells. Here, we showed that our extracted Mtb-HAg can markedly heighten the toxicity of γδ T cells, and that BTN3A represents a potential target for regulating γδ T cell function. Previously, it was unclear whether BTN3A also plays a pivotal role in the presentation and recognition of the peptide antigens used in our study, which could further enhance their potential efficacy. After administering the anti-BTN3A1 antagonist 103.1 mAb, we observed a significant reduction in both early activation markers and the release of cytotoxic factors from γδ T cells compared to that in cells treated only with Mtb-HAg. This suggests that BTN3A1 plays a crucial, positive regulatory role in Mtb-HAg-induced activation of γδ T cells and the enhancement of their cytotoxicity.

Other studies have demonstrated that the recognition of Mtb-derived peptide antigens by γδ T cells is primarily dependent on γδ TCR, a process that requires direct contact with antigen-presenting cells and signal transduction through this receptor. This mechanism operates independently of MHC I or II-mediated antigen presentation (70). Early research demonstrated that FITC-labeled Mtb-HAg and its purified components exhibited significant competitive inhibition with a PE-conjugated anti-TCR γδ monoclonal antibody during immunofluorescence staining of peripheral γδ T cells. Confocal microscopy further revealed that the FITC signal displayed substantial co-localization with PE-labeled γδ TCR fluorescence (76). Subsequent phage display screening targeting the CDR3 sequences of Mtb-HAg-expanded Vδ2+ T cells identified four mycobacterial protein-homologous peptides (77), providing direct evidence that γδ TCR mediates Mtb-HAg recognition. While current research on γδ T cell antigen recognition predominantly focuses on non-peptidic antigens (e.g., pAgs), the mechanisms governing peptide antigen recognition are still not well understood. Interestingly, although Mtb-HAg activates γδ T cells without requiring internalization, BTN3A1 antagonism significantly attenuated this activation. In this experiment, we observed that the activation of γδ T cells by Mtb-HAg also requires the involvement of BTN3A1; however, the detailed mechanism of this interaction was not explored, which is a limitation of this study. Future investigations could delve into the signaling pathways by constructing and transfecting point-mutated Vδ2-TCR recombinants. Meanwhile, a limitation of our blocking experiments is the absence of an isotype control antibody group. While our results show a clear and significant inhibitory effect using a well-characterized BTN3A-specific antibody compared to a no-antibody control, future studies should include isotype controls to conclusively exclude any potential non-specific Fc-mediated effects.

Our previous studies have confirmed that Mtb-HAg and its purified protein components can stimulate γδ T cells to secrete IFN-γ and TNF-α (17). These cytokines are also involved in regulating the survival and proliferation of intracellular pathogens (78, 79). There is also evidence that granulysin delivers granzymes into bacteria, exerting lethal effects and playing a critical role in killing Mtb (52, 80, 81). This experiment has not yet further investigated whether there are synergistic or additive effects between soluble cytokines (such as TNF-α and IFN-γ) and cytotoxic granule (such as GzmB, PFP, and GNLY). The limitations of the aforementioned experimental design also hinder an in-depth analysis of the comprehensive antibacterial effects of Mtb-HAg-activated γδ T cells, which should be further refined in subsequent studies.

In conclusion, our experimental results demonstrate that Mtb-HAg can effectively induce the activation of γδ T cells and enhance their cytolytic activity, which inhibits the growth of intracellular Mtb when cultured in contact with infected cells. Notably, this effect can be blocked by the anti-BTN3A1 antagonist 103.1 mAb.

## Data Availability

The datasets presented in this study can be found in online repositories. The names of the repository/repositories and accession number(s) can be found in the article/**Supplementary Material**.

## Supplementary material

Supplementary Figure 1 Electrophoretic identification and functional verification of Mtb-HAg. **(A)** SDS-PAGE analysis of Mtb-HAg. M: protein marker; 1: Mtb-HAg. **(B)** Flow cytometric analysis of γδ T cell proliferation in peripheral blood mononuclear cells (PBMCs) stimulated by Mtb-HAg. **(C)** Statistical analysis of γδ T cell proliferation (****P* < 0.001).

Supplementary Figure 2 Flow cytometric analysis of the efficiency of magnetic bead sorting for γδ T cells.

Supplementary Figure 3 Microscopic analysis of the cytotoxic effect of γδ T cells on infected THP-1-derived macrophages.

Supplementary Figure 4 Gating strategy for flow cytometry analysis.

Supplementary Table 1 Transcriptome overview.
