# Supplementary figures and images for: The mechanism regulating the cytotoxicity of γδ T cells activated by mycobacterium tuberculosis heat-resistant antigen based on RNA-seq analysis
Source: Front Immunol. 2026 Jan 28;17:1707304. doi: 10.3389/fimmu.2026.1707304 (PMC12891225; doi:10.3389/fimmu.2026.1707304)

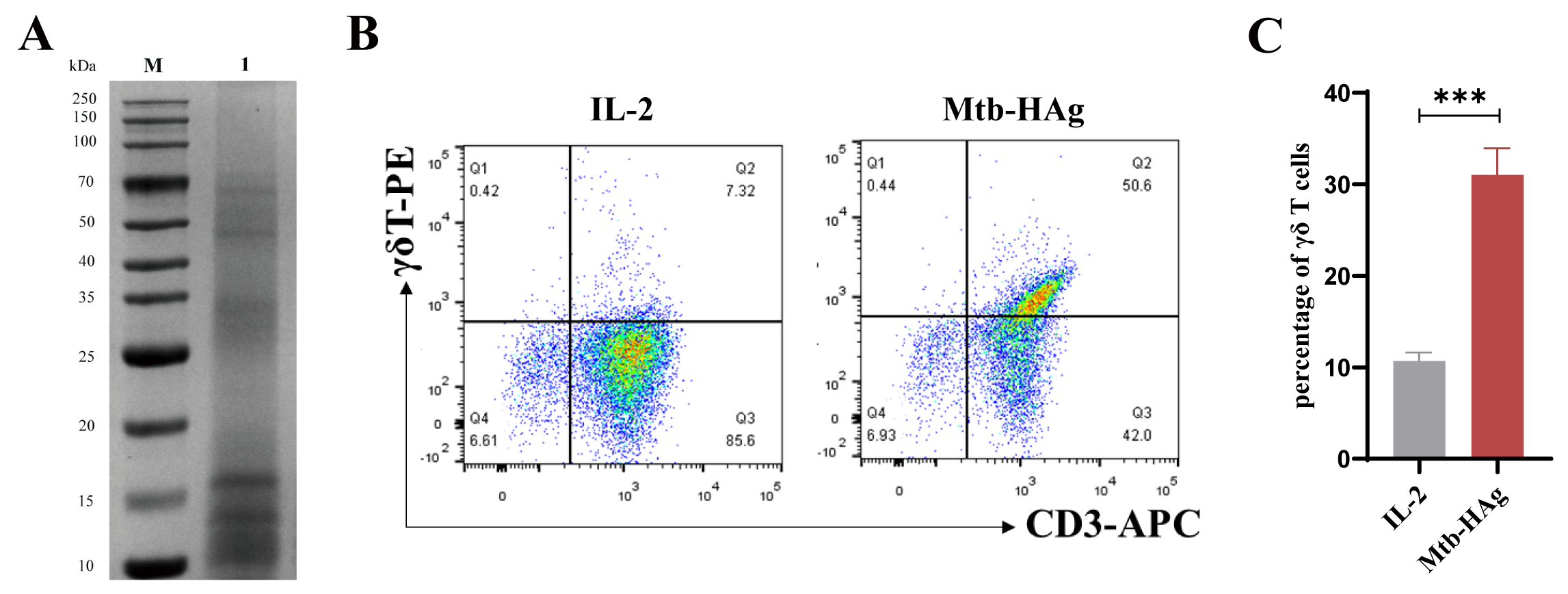

Supplement: Supplementary Figure 1 — Electrophoretic identification and functional verification of Mtb-HAg. (A) SDS-PAGE analysis of Mtb-HAg. M: protein marker; 1: Mtb-HAg. (B) Flow cytometric analysis of γδ T cell proliferation in peripheral blood mononuclear cells (PBMCs) stimulated by Mtb-HAg. (C) Statistical analysis of γδ T cell proliferation (***P < 0.001). [file Image1.tif]

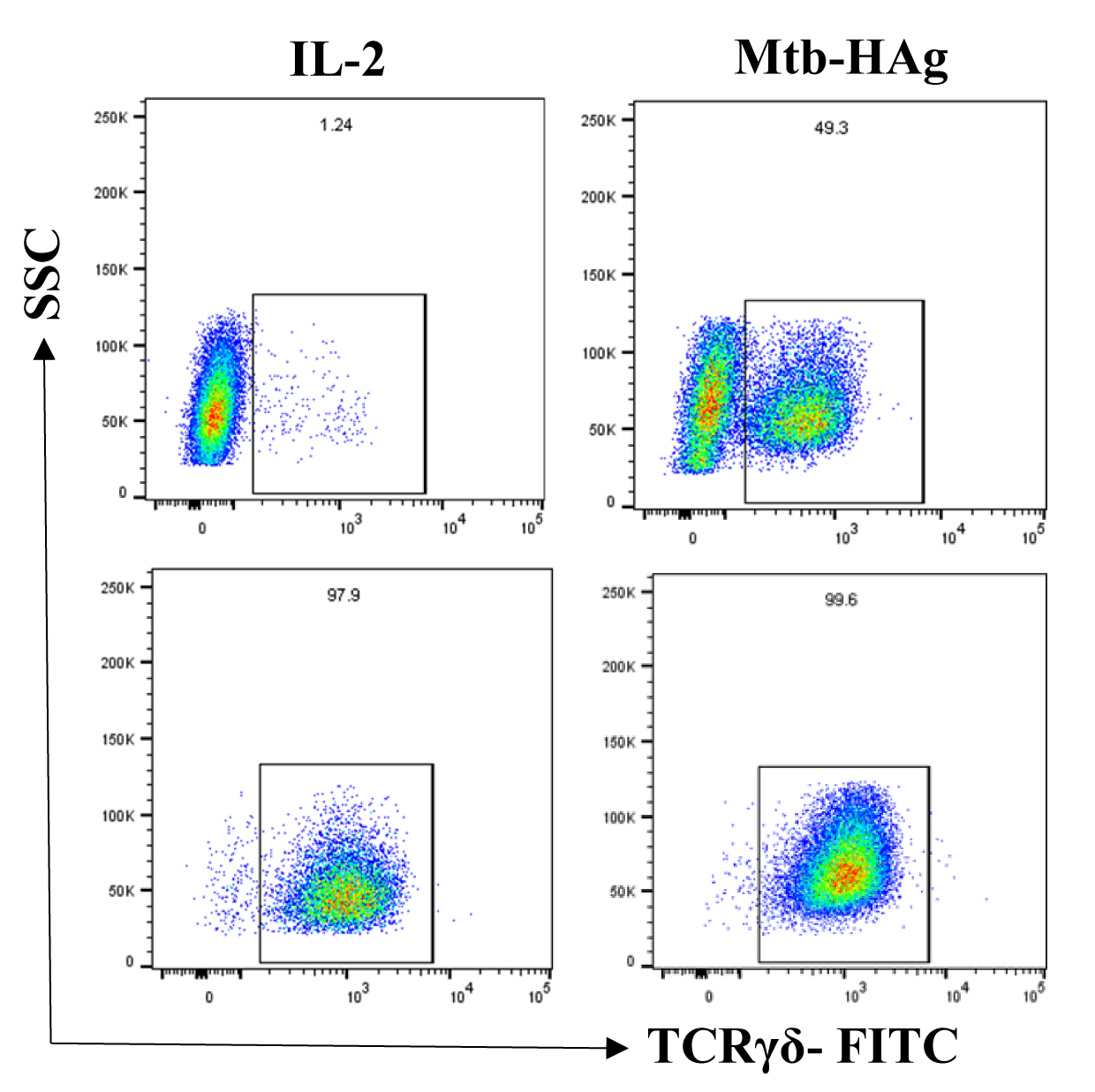

Supplement: Supplementary Figure 2 — Flow cytometric analysis of the efficiency of magnetic bead sorting for γδ T cells. [file Image2.tif]

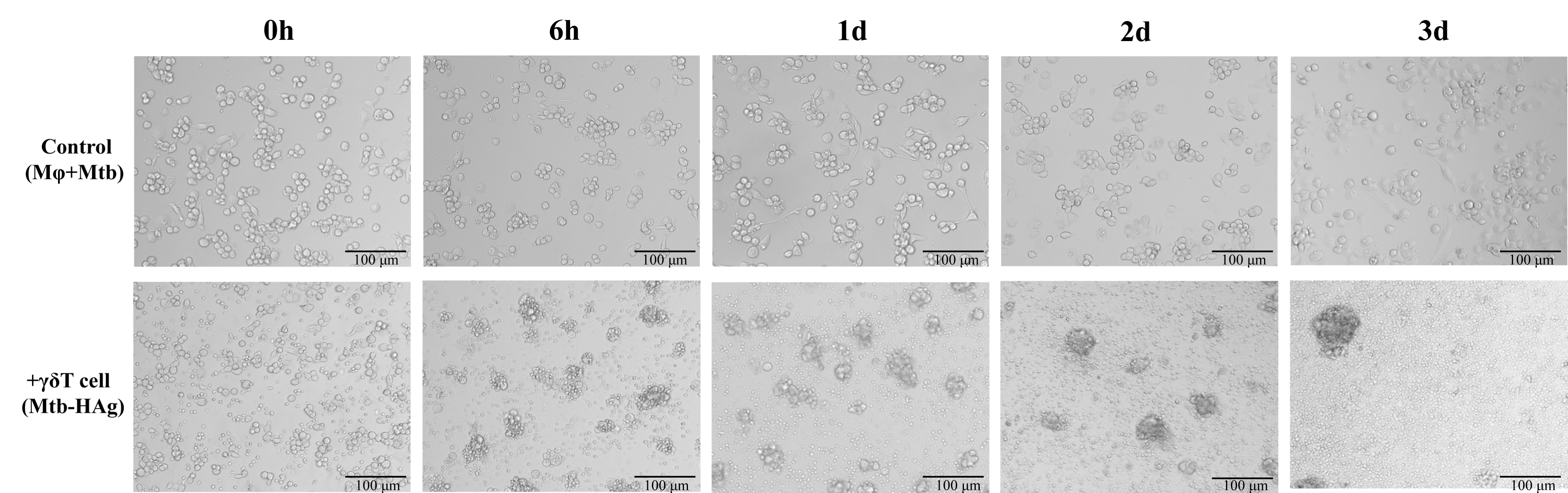

Supplement: Supplementary Figure 3 — Microscopic analysis of the cytotoxic effect of γδ T cells on infected THP-1-derived macrophages. [file Image3.tif]

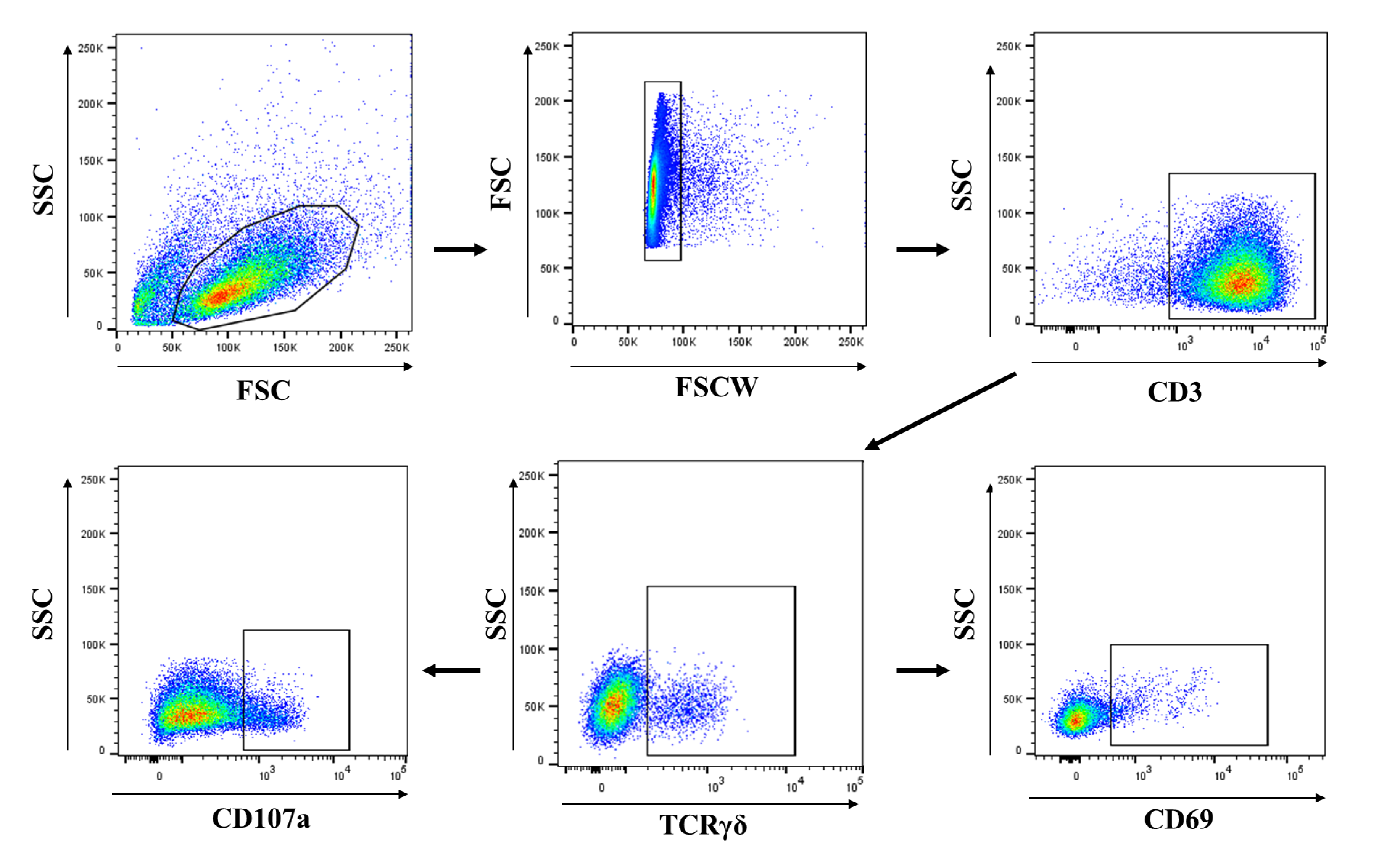

Supplement: Supplementary Figure 4 — Gating strategy for flow cytometry analysis. [file Image4.tif]
